# Supplementary material for: Single-Cell RNA Sequencing Analysis for Oncogenic Mechanisms Underlying Oral Squamous Cell Carcinoma Carcinogenesis with Candida albicans Infection
Source: Int J Mol Sci. 2022 Apr 27;23(9):4833. doi: 10.3390/ijms23094833 (PMC9104272; doi:10.3390/ijms23094833)
Supplement: Supplementary file 1 [file ijms-23-04833-s001.zip › ijms-1675046-supplementary.pdf]

| Group | Lesion location | Lesion type |
|---|---|---|
| **Normal tissue** | Gingival mucosa | Normal tissue |
| **OPMD with *Candida albicans* infection** | Buccal mucosa | Squamous hyperplasia |
| **OSCC without *Candida albicans* infection** | Buccal mucosa | Squamous cell carcinoma |
| **OSCC with *Candida albicans* infection** | Gingival mucosa | Squamous cell carcinoma |

Table S1: Lesion locations and types of patient specimens.

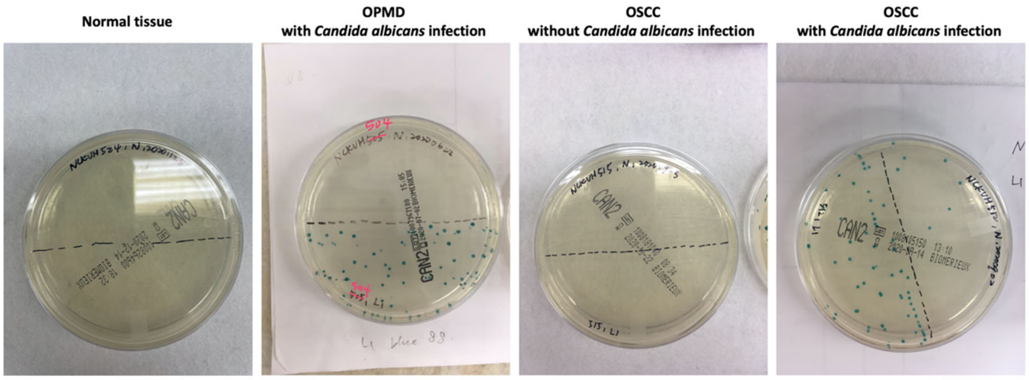


Figure S1: Candidiasis detection. CHROMagar Candida was used to detect whether patients were infected with *Candida albicans*. Green colony formation was recorded as a *C. albicans*-positive case.

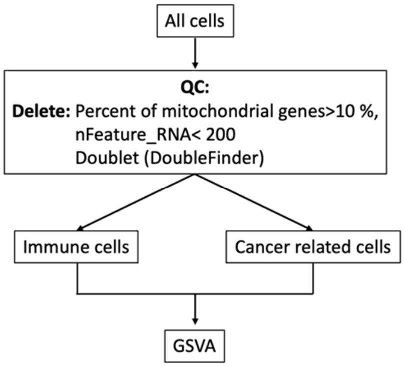


| Groups | Cell-ranger output cell number-Preset parameters | Cell-ranger output cell number-Forced output of 10,000 cells |
|---|---|---|
| Normal tissue | 7477 | - |
| OPMD with *Candida albicans* infection | 10045 | - |
| OSCC without *Candida albicans* infection | 3016 | 10000 |
| OSCC with *Candida albicans* infection | 2089 | 10000 |

| Groups | Normal tissue | OPMD with *Candida albicans* infection | OSCC without *Candida albicans* infection | OSCC with *Candida albicans* infection |
|---|---|---|---|---|
| Raw data | 7477 | 10045 | 10000 | 10000 |
| Percent.mt< 10 %, nFeature> 200 | 6345 | 7363 | 4837 | 2791 |
| After Doublet Finder | 5947 | 5819 | 4195 | 2472 |

Figure S2: Flow of quality control. Cells with >10% mitochondrial genes, <200 nFeature_RNA, and doublets were excluded.

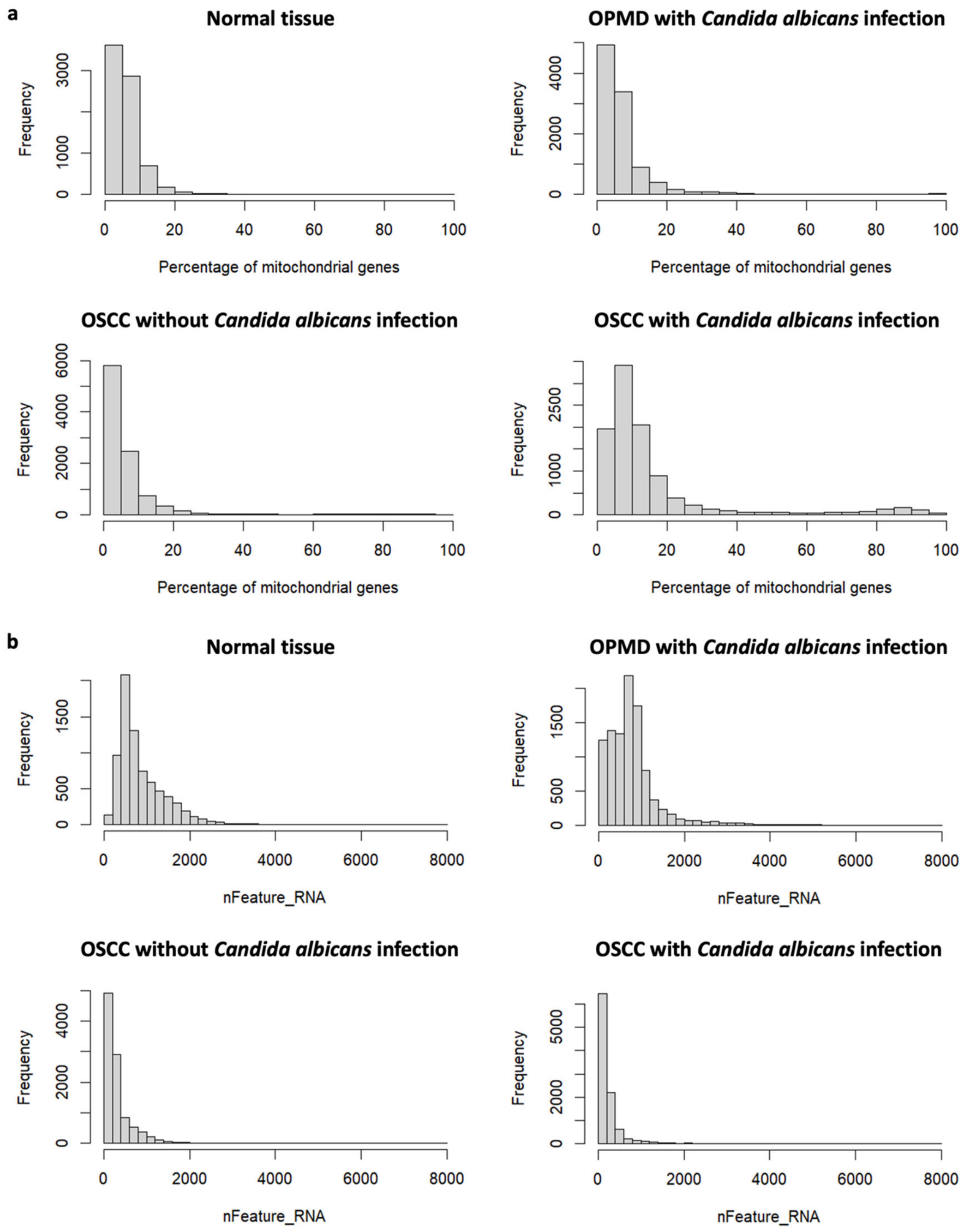


Figure S3: Performance of each group after quality control: (**a**) percentage of mitochondrial genes and (**b**) gene number in the cells.

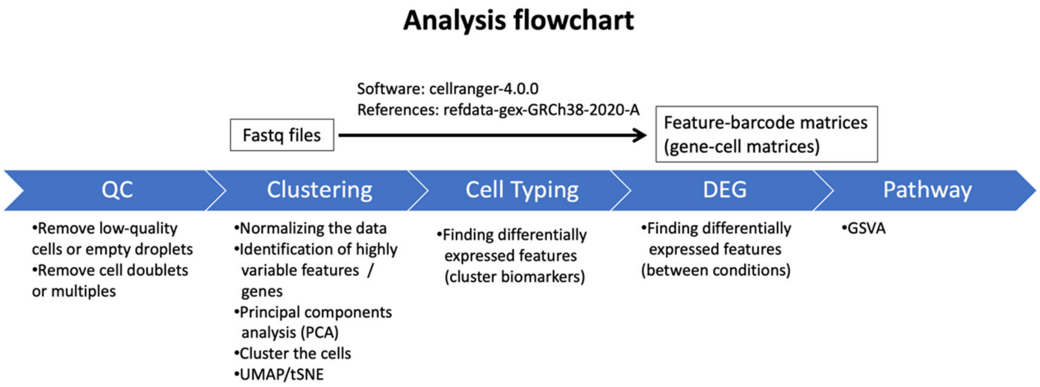


Figure S4: Analysis flow after next-generation sequencing.

| Cluster | 0 | 1 | 2 | 3 | 4 | 5 | 6 | 7 | 8 | 9 | 10 | 11 | 12 |
|---|---|---|---|---|---|---|---|---|---|---|---|---|---|
| Cell type | Fibroblast | Fibroblast | Fibroblast | Fibroblast | Epithelial | Fibroblast | Endothelial | Fibroblast | Endothelial | Epithelial | Others | Endothelial | Epithelial |
| Normal tissue | 29 % | 21 % | 0 % | 20 % | 2 % | 11 % | 5 % | 5 % | 0 % | 1 % | 4 % | 2 % | 0 % |
| OPMD with *Candida albicans* infection | 0 % | 2 % | 1 % | 0 % | 1 % | 32 % | 8 % | 16 % | 31 % | 1 % | 9 % | 0 % | 0 % |
| OSCC without *Candida albicans* infection | 0 % | 0 % | 59 % | 0 % | 0 % | 0 % | 11 % | 9 % | 15 % | 0 % | 1 % | 3 % | 0 % |
| OSCC with *Candida albicans* infection | 0 % | 0 % | 5 % | 0 % | 42 % | 0 % | 8 % | 0 % | 3 % | 20 % | 3 % | 7 % | 11 % |

| Cluster | 0 | 1 | 2 | 3 | 4 | 5 | 6 | 7 | 8 | 9 | 10 | 11 | 12 | 13 | 14 |
|---|---|---|---|---|---|---|---|---|---|---|---|---|---|---|---|
| Cell type | T-Cell | T-Cell | T-Cell | B-cell | T-Cell | Macrophage | B-cell | Others | Macrophage | T-cell | Macrophage | Neutrophils | Mast cell | Others | Others |
| Normal tissue | 41 % | 17 % | 8 % | 13 % | 1 % | 0 % | 8 % | 6 % | 2 % | 2 % | 0 % | 0 % | 3 % | 0 % | 0 % |
| OPMD with *Candida albicans* infection | 26 % | 27 % | 19 % | 13 % | 1 % | 1 % | 9 % | 1 % | 0 % | 2 % | 0 % | 0 % | 0 % | 0 % | 0 % |
| OSCC without *Candida albicans* infection | 13 % | 17 % | 9 % | 3 % | 33 % | 2 % | 1 % | 9 % | 8 % | 0 % | 0 % | 0 % | 0 % | 3 % | 0 % |
| OSCC with *Candida albicans* infection | 2 % | 2 % | 4 % | 2 % | 2 % | 57 % | 2 % | 5 % | 1 % | 0 % | 9 % | 9 % | 0 % | 0 % | 5 % |

Table S2. Cell ratios of the clusters within the four groups.

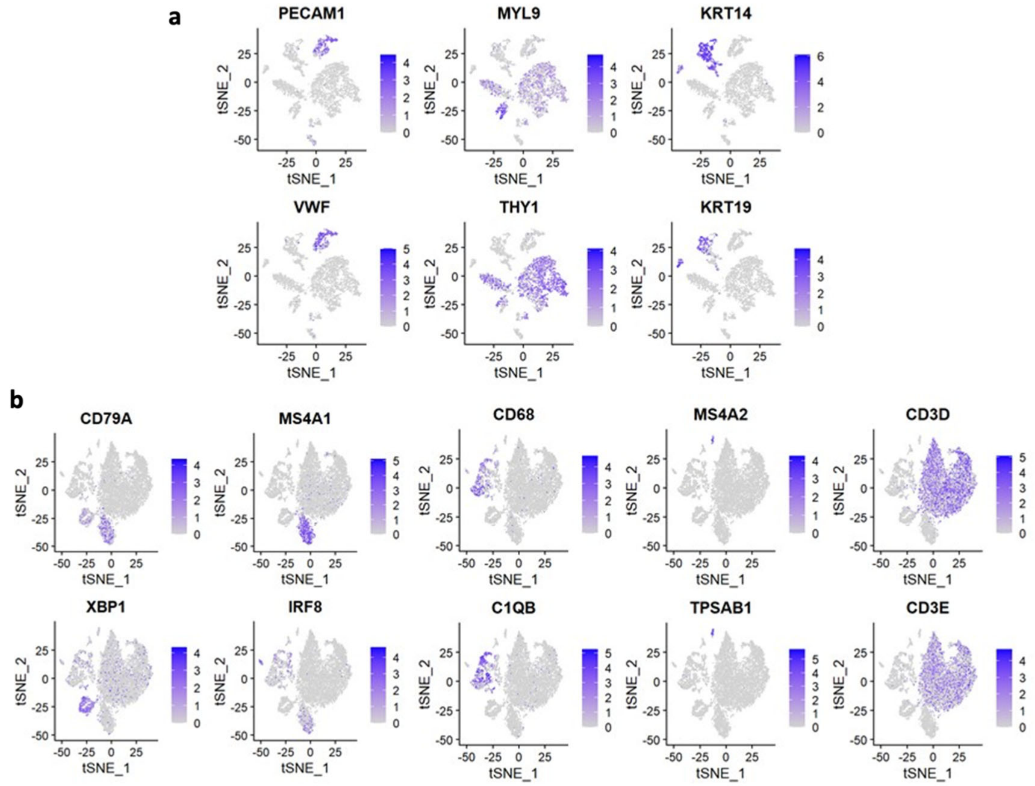


Figure S5: t-SNE for specific biomarkers distribution in the clusters. (a) PECAM1 and VWF are the specific biomarkers of endothelial cells, MYL9 and THY1 are those of fibroblast cells, and KRT14 and KRT19 are those of epithelial cells. (**b**) CD79A, XBP1, MS4A1, and IRF8 are the specific biomarkers of B cells; CD68 and C1QB are those of macrophages; MS4A2 and TPSAB1 are those of mast cells; and CD3D and CD3E are those of T cells.

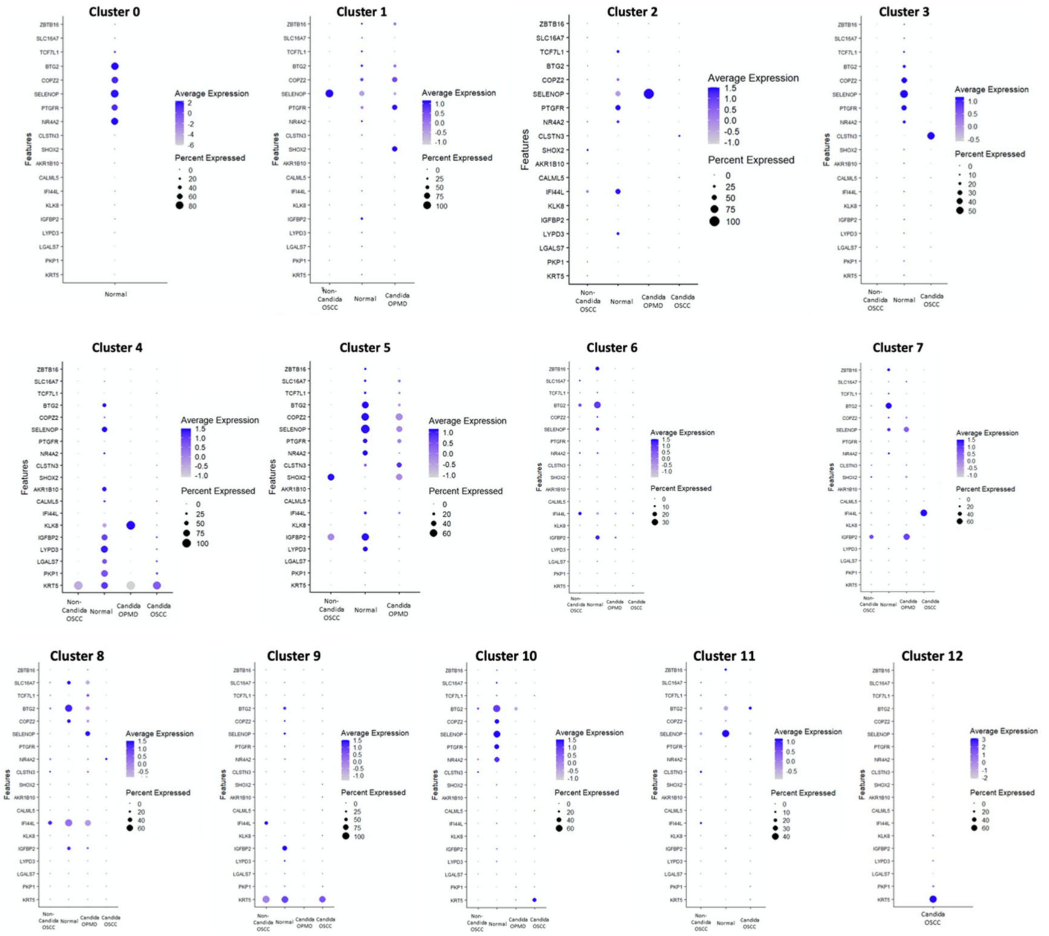


Figure S6: Dot diagrams presenting genes involved in KRAS_SIGNALING_DN. Expression of genes involved in the KRAS signaling pathway in 13 clusters within the four groups is illustrated. The average gene expression levels and percentages of cells in the four groups are indicated using different colors and sizes of the dots.

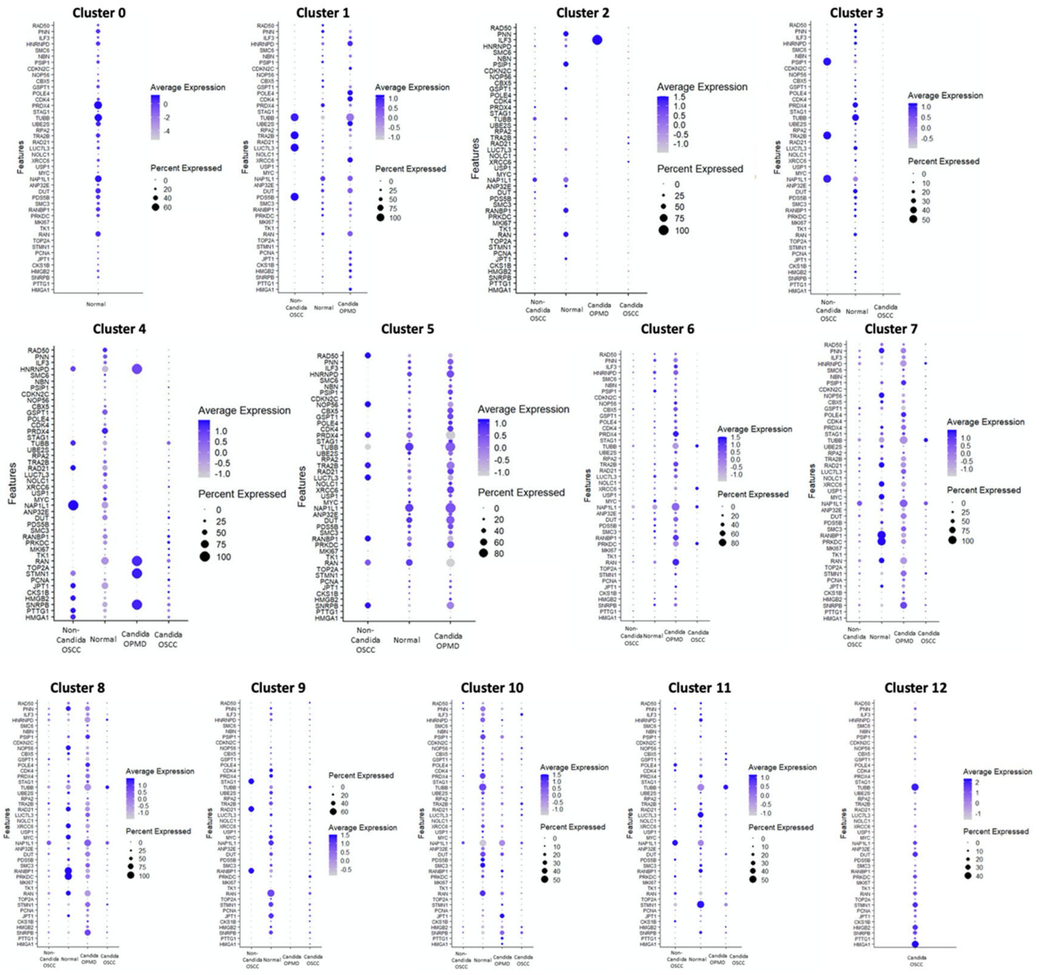


Figure S7: Dot diagrams presenting genes involved in the E2F_TARGETS. Expression of E2F target genes in 13 clusters within the four groups are illustrated. The average gene expression levels and percentages of cells in the four groups are indicated using different colors and sizes of the dots.

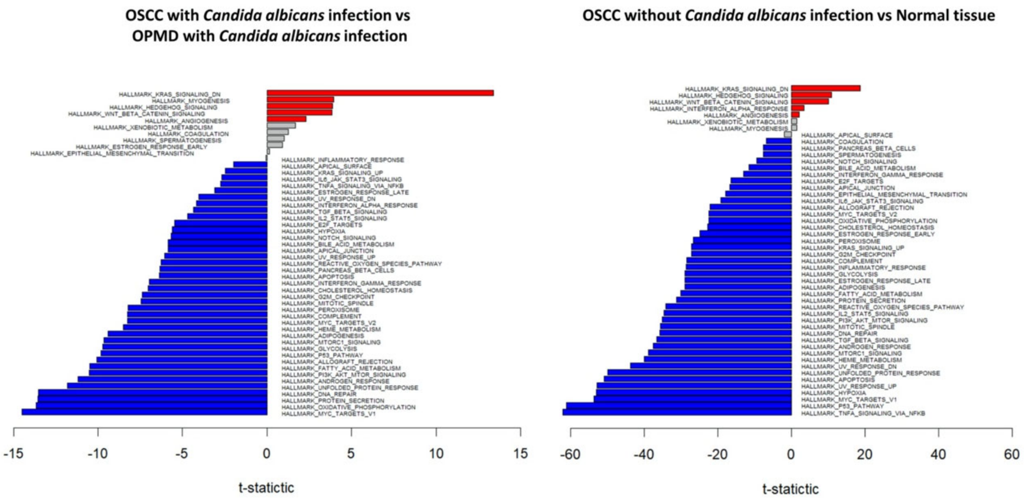


Figure S8: GSVA of regulatory pathways in (**a**) OSCC tissue with *Candida albicans* infection and OPMD with *Candida albicans* infection. Red and blue bars indicate that gene expression was significantly increased in OSCC tissue with *C. albicans* infection compared with OPMD lesion with *Candida albicans* infection and OPMD lesion with *Candida albicans* infection compared with OSCC tissue with *C. albicans* infection. (**b**) OSCC tissue without *Candida albicans* infection and normal tissue. Red and blue bars indicate that gene expression was significantly increased in OSCC tissue without *C. albicans* infection compared with normal tissue and normal tissue compared with OSCC tissue without *C. albicans* infection
